# Disease-linked mutations in factor H reveal pivotal role of cofactor activity in self-surface–selective regulation of complement activation

**DOI:** 10.1074/jbc.M117.795088

**Published:** 2017-06-21

**Authors:** Heather Kerr, Edwin Wong, Elisavet Makou, Yi Yang, Kevin Marchbank, David Kavanagh, Anna Richards, Andrew P. Herbert, Paul N. Barlow

**Affiliations:** From the Schools of Chemistry and Biological Sciences, Joseph Black Building, University of Edinburgh, David Brewster Road, Edinburgh EH9 3FJ, Scotland, United Kingdom

**Keywords:** complement system, erythrocyte, innate immunity, kidney, mutagenesis, recombinant protein expression, Streptococcus, surface plasmon resonance (SPR), CCP module, multiple domain

## Abstract

Spontaneous activation enables the complement system to respond very rapidly to diverse threats. This activation is efficiently suppressed by complement factor H (CFH) on self-surfaces but not on foreign surfaces. The surface selectivity of CFH, a soluble protein containing 20 complement-control protein modules (CCPs 1–20), may be compromised by disease-linked mutations. However, which of the several functions of CFH drives this self-surface selectivity remains unknown. To address this, we expressed human CFH mutants in *Pichia pastoris*. We found that recombinant I62-CFH (protective against age-related macular degeneration) and V62-CFH functioned equivalently, matching or outperforming plasma-derived CFH, whereas R53H-CFH, linked to atypical hemolytic uremic syndrome (aHUS), was defective in C3bBb decay-accelerating activity (DAA) and factor I cofactor activity (CA). The aHUS-linked CCP 19 mutant D1119G-CFH had virtually no CA on (self-like) sheep erythrocytes (*E*_S_) but retained DAA. The aHUS-linked CCP 20 mutant S1191L/V1197A-CFH (LA-CFH) had dramatically reduced CA on *E*_S_ but was less compromised in DAA. D1119G-CFH and LA-CFH both performed poorly at preventing complement-mediated hemolysis of *E*_S_. PspCN, a CFH-binding *Streptococcus pneumoniae* protein domain, binds CFH tightly and increases accessibility of CCPs 19 and 20. PspCN did not improve the DAA of any CFH variant on *E*_S_. Conversely, PspCN boosted the CA, on *E*_S_, of I62-CFH, R53H-CFH, and LA-CFH and also enhanced hemolysis protection by I62-CFH and LA-CFH. We conclude that CCPs 19 and 20 are critical for efficient CA on self-surfaces but less important for DAA. Exposing CCPs 19 and 20 with PspCN and thus enhancing CA on self-surfaces may reverse deficiencies of some CFH variants.

## Introduction

**Figure 1.**
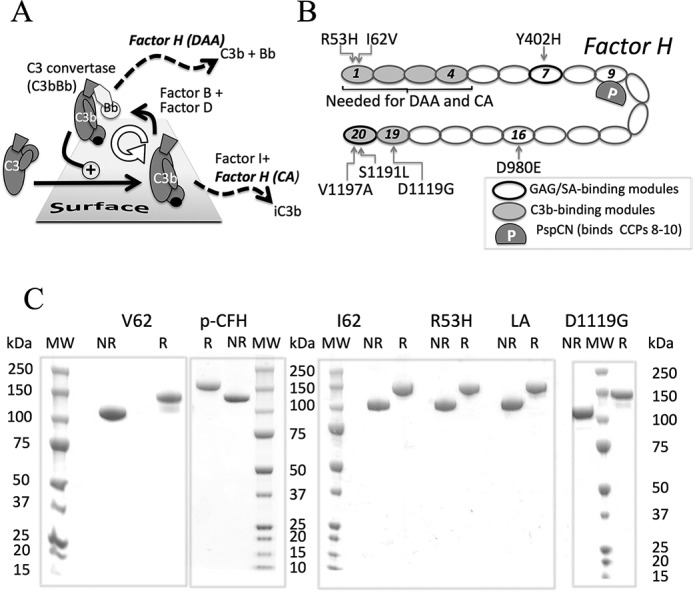
**CFH and its roles in regulating complement.** *A*, amplification of C3b via C3bBb. CFH accelerates irreversible decay of C3bBb and is a cofactor for CFI that cleaves C3b to iC3b. *B*, schematic of CFH highlighting mutations investigated in this study. CFH consists exclusively of 20 CCPs, shown as *ovals*. C3b-binding sites (*shaded*) are located in N-terminal and C-terminal regions. The C3b-binding site in CCPs 19 and 20 also binds iC3b, C3dg, and C3d; it is at least partially cryptic and is exposed when CFH binds PspCN (*P*) (a domain from a bacterial virulence factor) or when CFH is on a self-surface. Self-surface recognition sites, which bind to GAGs and SAs, reside in CCPs 7 and 20. CCPs 1–4 are necessary and sufficient (in fluid phase) for DAA and CA. *C*, five deglycosylated recombinant proteins used herein (V62-CFH, I62-CFH, R53H-CFH, D1119G-CFH, and LA-CFH) along with CFH purified from pooled plasma (*p-CFH*) were subjected to SDS-PAGE under both reducing (*R*) and non-reducing (*NR*) conditions and stained with Coomassie Blue. *MW*, molecular weight markers.

The complement system provides antibody-independent first-line immune defense. It also augments antibody-mediated and cellular immunity (1). In addition, complement contributes to clearance of by-products of cellular metabolism and cell turnover (2). The cleavage of C3, a thioester-containing serum protein, into C3a and C3b, is pivotal to all three pathways of complement activation. In the alternative pathway, C3 is cleaved by C3bBb, a short-lived complex of C3b and Bb, a serine protease. In a Mg^2+^-dependent process, C3bBb-generated C3b binds to complement factor B (CFB),^3^ a proenzyme, whereupon CFB is cleaved by complement factor D (CFD) to yield more C3bBb. Thus, a C3b amplification loop is generated (3) (Fig. 1*A*). Transiently, nascent C3b can utilize its freshly activated thioester to covalently attach to surfaces (4). Here, unless regulated, C3b is amplified, forms clusters, functions as an opsonin, and triggers further steps in the complement cascade, including C5 cleavage, C5a release, and formation of membrane attack complexes.

A set of plasma proteins calibrates C3b amplification. Properdin (5) stabilizes C3bBb, whereas complement factor H (CFH) (6–8) (Fig. 1*B*) destabilizes it; this is called decay-accelerating activity (DAA). CFH also has cofactor activity (CA) for cleavage of C3b by complement factor I (CFI). The product, iC3b, cannot bind CFB but is an opsonin in its own right (9). iC3b may be further degraded to C3dg and, ultimately, to C3d. C3d(g) remains covalently bound to the surface for an extended period of time and thereby marks sites of historical complement activation and regulation. As a soluble circulating complement regulator, CFH suppresses spontaneous complement activation in fluid phase that is considered “futile” and that consumes C3 and factor B. Critically, CFH efficiently suppresses C3b amplification on self-surfaces (10) but not on foreign ones. Other host complement regulators (11), which also exhibit DAA and/or CA, are permanently attached to cell surfaces. Together, the complement regulators ensure that the opsonizing, inflammatory, and cytolytic consequences of complement activation manifest on targets rather than on healthy self-surfaces. CFH-related proteins 1–5 (CFHR-1 to -5) (12) provide additional regulatory sophistication. Some of them antagonize CFH by competing with it for binding to targets including C3b (13, 14).

Inherited variations in membrane cofactor protein (MCP, CD46), CFB, C3, CFI, CFHRs, and, in particular, CFH are linked to diseases (15, 16). Numerous disease-related sequence variations were identified in CFH, which consists exclusively of 20 complement-control protein modules (CCPs). The SNP, H402Y, within the seventh CCP (CCP 7) is linked to age-related macular degeneration (AMD) (17–20) and the kidney disorder, C3 glomerulopathy (C3G) (21); H402 is the at-risk allele. The SNP, I62V, in CFH CCP 1 is also linked to AMD (20, 22); Ile-62 is protective. Deletion of the *CFHR-1* and *CFHR-3* genes decreases the risk of immunoglobulin A nephropathy and AMD (23–26) but predisposes to systemic lupus erythematosus and atypical hemolytic uremic syndrome (aHUS), a form of thrombotic micoangiopathy leading to end-stage renal failure (27). Most mutations found in aHUS patients cluster in the CFH C-terminal CCPs 19 and 20 (28). Other potentially disease-linked sequence variations occur elsewhere in CFH (29–31).

Penetrance is low for many of the CFH mutations linked to diseases. Variation in the genes for other complement proteins and environmental factors may also contribute to disease risk (32). The complexity of complement modulation underlines the need to explore functional effects of each potentially disease-causing CFH variant. However, the scarcity of pure CFH samples is limiting (22, 33). Most patients are heterozygous for mutations that occur on a polymorphic background. Recombinant production of full-length CFH, a single polypeptide containing 40 disulfides (two per CCP), is challenging (34–36). In only one case (36) were yields sufficient for rigorous sample authentication.

Many studies have tried to recapitulate disease-linked mutations in more easily produced CFH truncations containing up to five CCPs (37–42). Indeed, most disease-linked variations occur within subsets of CCPs, namely CCPs 1–4, CCP 7, or CCPs 19 and 20, which coincide with functional sites (43) (Fig. 1*B*). For example, a fragment consisting of CFH CCPs 1–4 (CFH 1–4) binds C3b and has CA (44) and DAA (45). CFH 19-20 has neither CA nor DAA but binds C3b, iC3b, and C3d(g) (46). Recombinant CFH 6–8 and CFH 19-20 bind glycosaminoglycans (GAGs) and sialic acids (SAs) found on self-surfaces (47). Recombinant CFH 19-20 binds well to C3d and thereby competes with full-length CFH for binding to C3b-decorated sheep erythrocytes (*E*_S_) (37), whereas disease-related mutations in CCPs 19 and 20 perturbed this ability (39).

Crucially, the use of small CFH truncations cannot capture the collaboration between multiple binding sites that characterizes CFH function in its biological setting. CFH probably undergoes a conformational adjustment (48–50) in a concerted process that involves engagement, via GAG-binding and SA-binding sites in CCPs 7 and 20, with self-surface markers (43). This is thought to allow the C3b/C3d(g)-binding sites located in CCPs 1–4 and CCPs 19 and 20 (47) to engage simultaneously with C3b molecules bound on a host surface (50). Crucially, the resultant CFH–C3b–GAG/SA complex is better at complement suppression than the CFH–C3b complex formed on a surface lacking GAGs and SAs. Whether this enhanced efficacy on self-surfaces is driven by higher DAA, higher CA, or both is unknown. Recombinant full-length CFH is needed to investigate these key self *versus* non-self discriminatory properties and to establish the phenotypic consequences of disease-related mutations.

Herein, we illustrate the value of producing recombinant full-length CFH for characterizing disease-linked sequence variations. After benchmark work on substitutions in N-terminal CCPs, we focused on potentially more revealing mutations in C-terminal CCPs (Fig. 1*B*). We investigated the activities of these CFH mutants on the man-made surfaces of surface plasmon resonance (SPR) chips and the foreign surface of rabbit erythrocytes (*E*_R_) as well as on the more hostlike surface of *E*_S_. We additionally investigated the functions of our mutants when in complex with PspCN. PspCN is a domain from a bacterial complement evasion protein that binds very tightly to CFH (51) and causes it to adopt a different conformation with enhanced regulatory efficacy (50). Binding to CFH of PspCN increases the accessibility of CCPs 19 and 20, as also probably occurs when CFH binds to a self-surface. Our studies suggest that CA, more than DAA, is boosted by interactions between the C-terminal region of CFH and self-surfaces.

## Results

### High-yield production of CFH variants in P. pastoris

We produced the two common allotypic CFH variants with Ile or Val at position 62 of the mature protein (I62-CFH or V62-CFH, respectively) in recombinant *P. pastoris*. We also produced the disease-related mutants, R53H-CFH (on Ile-62 background), D1119G-CFH (on Val-62 background), and S1191L/V1197A-CFH (henceforth LA-CFH) (on Ile-62 background). The *P. pastoris*-secreted proteins were harvested prior to endoglycosidase treatment to cleave mannose-rich and hence non-native *N*-glycans back to the first GlcNAc residues. Following purification, homogeneity was assessed using SDS-PAGE (Fig. 1*C*). The absence of extra bands in the gel, under both reducing and non-reducing conditions, indicates minimal proteolytic cleavage. Note that cleavage within a CCP might have been evident only in reduced samples due to the interlaced disulfide bonds within each CCP (*i.e.* Cys^(i)^ to Cys^(iii)^ and Cys^(ii)^ to Cys^(iv)^) (52). All proteins were prepared in multiple 10-mg quantities.

### V62-CFH and I62-CFH have similar biological activities

**Figure 2.**
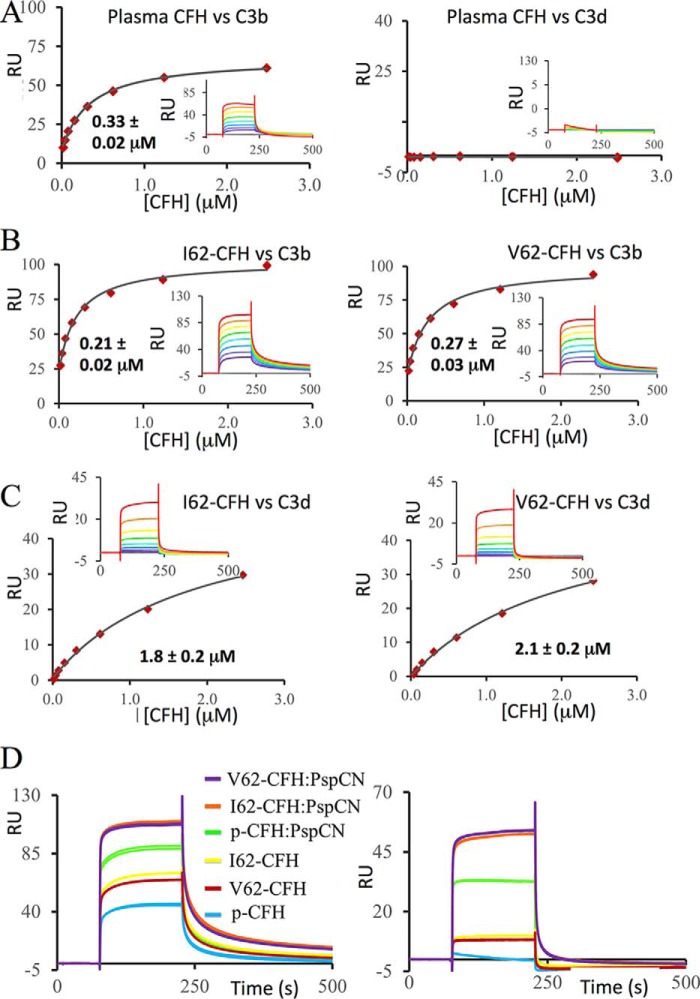
**C3b and C3d binding of plasma-purified CFH, I62-CFH, and V62-CFH.** All data shown here were collected in one SPR experiment on the same C1-sensor chip. Care was taken to ensure that the CFH samples were all of equivalent quality and concentration. *A*, 2-fold dilution series (from 2.5 to 0.019 μm) of CFH from plasma were flowed in duplicate over amine-coupled C3b (*left*) or C3d (*right*). Equilibrium plots were used in an attempt to estimate *K_D_* values (Table 1), but no affinity measurement could be made for C3d (*inset*, background-subtracted sensorgrams). *B*, 2-fold dilution series (from 2.5 to 0.019 μm) of I62-CFH (*left*) or V62-CFH (*right*) were flowed in duplicate over amine-coupled C3b, and *K_D_* values were estimated as above. Two further experiments comparing V62-CFH with plasma CFH indicated similar binding to C3b. *C*, an equivalent set of experiments to *B* but with C3d instead of C3b. Multiple replicates confirmed the discrepancy in C3d binding between recombinant CFH and plasma CFH. *D*, sensorgrams for I62-CFH, V62-CFH, or plasma CFH (*p-CFH*) all at 0.6 μm, with or without PspCN (at 1.2 μm), flowed over C3b (*left*) or C3d (*right*). Very similar PspCN-induced enhancements of both plasma CFH and recombinant CFH binding to C3b and C3d were observed in multiple experiments*; K_D_* values were estimated for I62-CFH alone *versus* I62-CFH–PspCN complex (see Table 1).

We compared binding to C3b of I62-CFH, V62-CFH, and CFH purified from human plasma (plasma CFH). Both recombinant protein samples were freshly produced, purified, and assayed in parallel. The plasma CFH sample was purified from pooled human plasma and contained no CFH-related proteins detectable by Coomassie Blue-stained SDS-PAGE (Fig. 1*C*). Aliquots were stored at −20 °C. An aliquot was freshly thawed for each experiment. The C3b sample (Complement Technology, Tyler, TX) had been stored at −80 °C and was also thawed immediately before use. The C3b was immobilized on a Biacore C1-sensor chip, using amine coupling. By flowing CFH solutions over amine-coupled C3b (Fig. 2, *A* and *B*), we obtained similar *K_D_* values (in the range of 0.21–0.33 μm) for all three proteins (Table 1).

**Table 1:** **Summary of SPR data**

Experiment	Variant	Ligand	Without PspCN				With PspCN			
			*K_D_* (μm)*^a^*	*R*_max_	*R*_offset_	χ^2^	*K_D_* (μm)	*R*_max_	*R*_offset_	χ^2^
I62-CFH *versus* V62-CFH *versus* plasma CFH	I62-CFH	C3b	0.21 ± 0.02	79.2	32.2	5.2				
	V62-CFH	C3b	0.27 ± 0.03	80.0	19.3	4.6				
	plasma-CFH	C3b	0.33 ± 0.02	59.6	8.0	0.6				
	I62-CFH	C3d	1.8 ± 0.2	50.6	0.5	0.3				
	V62-CFH	C3d	2.1 ± 0.2	52.2	0.0	0.2				
	plasma-CFH	C3d	high							
I62-CFH *versus* mutants, with/without PspCN (Expt. 1)*^b^*	I62-CFH	C3b	1.2 ± 0.2	175	34.8	5.2	0.50 ± 0.07	266	63.5	31
	R53H-CFH	C3b	1.8 ± 0.3	217	42.9	6.5	1.0 ± 0.1	310	75.3	13
	LA-CFH	C3b	0.83 ± 0.10	181	46.6	8.3	0.70 ± 0.07	249	91.2	16
I62-CFH *versus* mutants, with/without PspCN (Expt. 2)	I62-CFH	C3b	1.0 ± 0.2	117	41.2	4.0	0.62 ± 0.08	132	55.6	5.8
	R53H-CFH	C3b	1.2 ± 0.1	152	41.1	3.7	0.58 ± 0.04	134	52.1	2.5
	LA-CFH	C3b	0.86 ± 0.11	146	45.7	6.6	0.60 ± 0.07	151	61.4	8.0

*^a^* Shown are the S.E. values for the fits of the data from each experiment.

*^b^* Expt., experiment.

We also compared binding of these three versions of CFH to C3d (Complement Technology) amine-coupled to a different flow cell on the same C1-sensor chip as used for the C3b-binding experiments (Fig. 2, *A* and *C*). Both I62-CFH and V62-CFH bound to immobilized C3d with *K_D_* values of ∼1.9 μm (Table 1). Conversely, binding of plasma CFH to C3d was, as observed previously (13, 49, 50), too weak to measure (Fig. 2*A*). The lack of binding between plasma CFH and C3d was previously attributed to lack of access to the C3d-binding site in CCPs 19 and 20. Indeed, premixing 0.6 μm plasma CFH with 1.2 μm PspCN transformed it into a C3d binder (Fig. 2*D* and Table 1), as seen previously (50). Premixing I62-CFH or V62-CFH with PspCN also enhanced their binding to both C3b and C3d (Fig. 2*D* and Table 1). For confirmation, two additional SPR-based experiments were performed on a different batch of I62-CFH, each on a different day with a different chip. In both experiments (Table 1), the *K_D_* of I62-CFH for C3b was approximately halved following preincubation (of I62-CFH) with a 2-fold molar excess of PspCN. Thus, both plasma CFH and recombinant CFH bind PspCN, via their central CCPs (50), and undergo a conformational change that renders the C-terminal modules more accessible to C3d.

**Figure 3.**
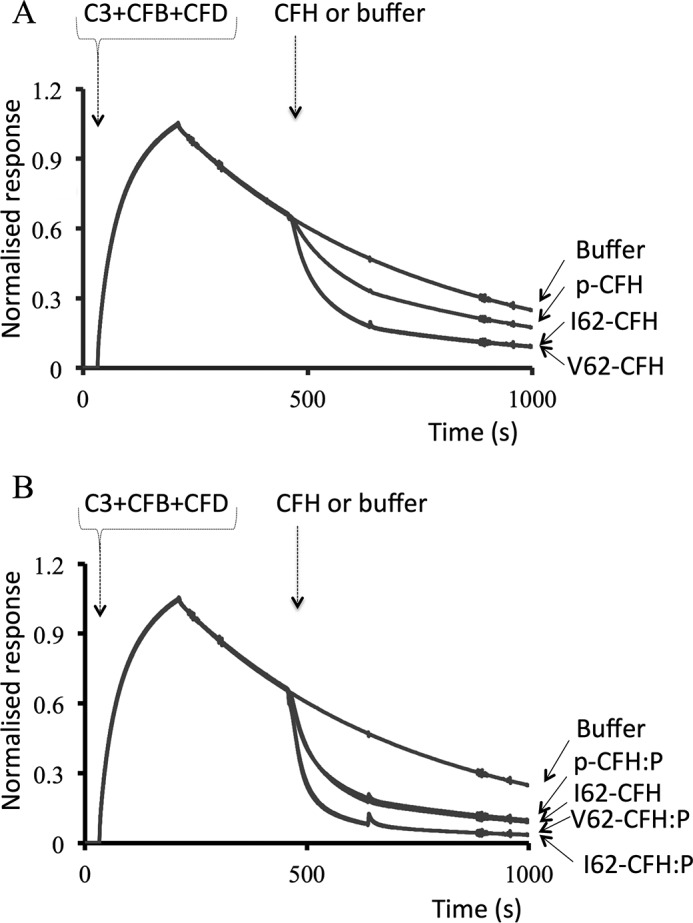
**Decay-accelerating activity of CFH on SPR sensor chips.** *A*, C3bBb was formed on a CM5-sensor chip by flowing C3, CFB, and CFD over an immobilized C3b “seed.” The C3bBb DAAs of 20 nm V62-CFH and 20 nm I62-CFH were compared with that of 20 nm plasma CFH (*p-CFH*). *B*, these experiments were repeated but with a 1:2 mixture of CFH and PspCN (*i.e. CFH:P*). The curve for I62-CFH from (*A*) (*i.e.* with no PspCN) is included for reference; note that I62-CFH and plasma-derived CFH–PspCN (*p-CFH:P*) have overlapping curves. The data shown are representative of an experiment performed four times.

In SPR-based assays on a CM5-sensor chip (Biacore), we compared the abilities of recombinant and plasma CFH to accelerate decay of the preassembled C3bBb, the alternative pathway C3 convertase. Both I62-CFH and V62-CFH, at 20 nm, had identical DAA in this assay, whereas 20 nm plasma CFH was less active (Fig. 3*A*). The presence of PspCN (at a 2:1 ratio to CFH) enhanced the DAA of I62-CFH and V62-CFH to identical extents (Fig. 3*B*). As expected, PspCN also enhanced the DAA of plasma CFH in this assay.

**Figure 4.**
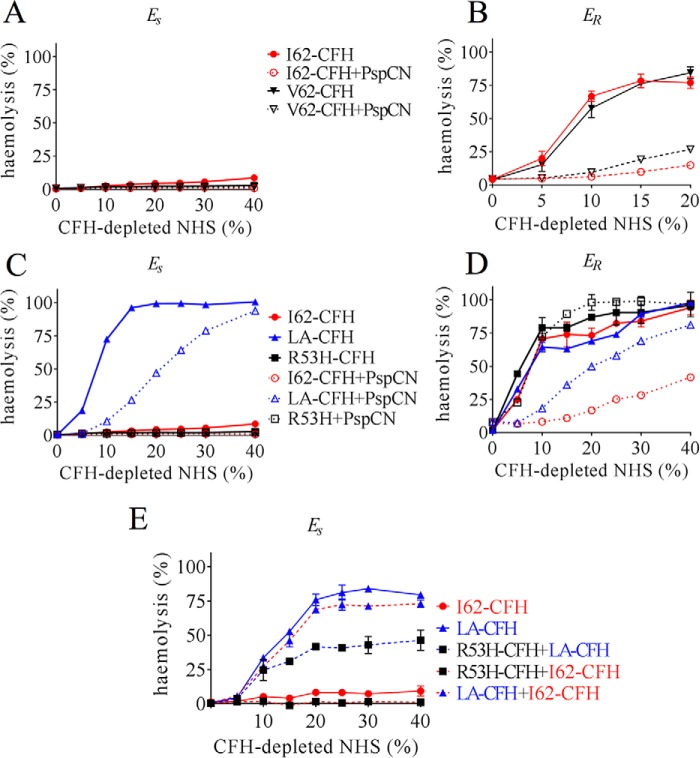
**Erythrocyte-protection assays.** *E*_S_ or *E*_R_ were incubated with CFH-depleted NHS at various percentages (v/v) that had been reconstituted with our recombinant CFH variants. *A*, reconstitution with 250 nm I62-CFH or 250 nm V62-CFH afforded protection of *E*_S_, with or without 500 nm PspCN. *B*, neither 1 μm I62-CFH nor 1 μm V62-CFH protected *E*_R_. PspCN (2 μm) enhances CFH protection in both cases. *C*, LA-CFH (500 nm), unlike 250 nm I62-CFH, offered little protection against *E*_S_ lysis. No lysis occurred in the presence of 250 nm R53-CFH. The addition of 500 nm PspCN to 250 nm LA-CFH (final concentrations) improved its protective properties. *D*, neither 1 μm LA-CFH (alone) nor 1 μm R53-CFH (with or without 2 μm PspCN) protected *E*_R_, but a mixture of 1 μm LA-CFH + 2 μm PspCN was more protective than LA-CFH alone. *E*, LA-CFH (500 nm) (unlike 500 nm I62-CFH) did not protect *E*_S_ from lysis; nor did a mixture of 250 nm LA-CFH + 250 nm I62-CFH. A mixture of 250 nm R53H-CFH + 250 nm I62-CFH was protective. A mixture of 250 nm R53H-CFH + 250 nm LA-CFH was more protective than 500 nm LA-CFH alone but less protective than R53-CFH alone (see *C*). Six independent measurements were made in *B*, whereas *A* and *C–E* are representative of two independent experiments. The mean and S.D. (*error bars*) for each measurement were calculated for all data sets (in most cases, error bars are obscured by the *symbol*).

Functional similarities between I62-CFH and V62-CFH extended to hemolysis protection assays. Upon exposure to increasing percentages of CFH-depleted normal human serum (NHS), *E*_S_ are susceptible to lysis by complement unless protected by added CFH. *E*_R_, in contrast, lack human-like SAs and are not effectively protected by human CFH. Both I62-CFH and V62-CFH proved equally effective (*e.g.* at 250 nm) in preventing *E*_S_ hemolysis (Fig. 4*A*) in this assay, whereas both were, as expected, equally ineffective, at 1 μm, in protecting *E*_R_ (Fig. 4*B*). Both I62-CFH and V62-CFH showed more ability to prevent *E*_R_ hemolysis when in complex with PspCN.

**Figure 5.**
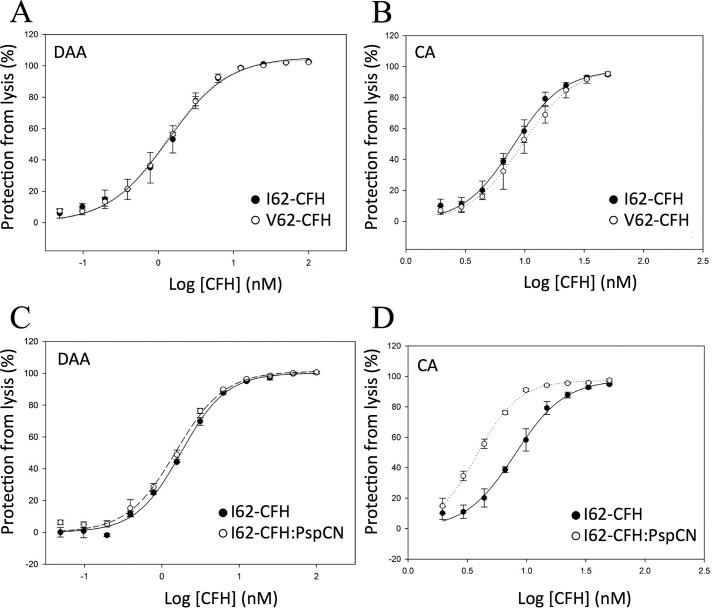
**Decay-accelerating and cofactor activities of I62-CFH and V62-CFH on *E*_S_.** *A*, activated *E*_S_ were preloaded with C3bBb. The C3bBb-decorated cells were exposed to a CFH concentration series, followed by a hemolysis-based estimate of remaining C3bBb. This allowed derivation of IC_50_ values for DAA on a hostlike cell surface. *B*, C3b-preloaded activated *E*_S_ surfaces were treated with CFI and a range of CFH concentrations, providing an estimate of the CA of CFH on a hostlike cell surface. *C*, the DAA assay used in *A* was repeated, but using I62-CFH mixed (1:2) with PspCN. The curve for I62-CFH alone is included for comparison. *D*, the CA assay used in *B* was repeated, but using I62-CFH mixed (1:2) with PspCN. The curve for I62-CFH alone is included for comparison. The mean and S.D. (*error bars*) of triplicate measurements are plotted, and sigmoidal curves were fitted using SigmaPlot. Each graph is representative of two experiments, except for *A* and *D*, which show data from single experiments.

In a more specific *E*_s_-surface assay (22), C3bBb was preassembled on *E*_S_, and the resultant C3bBb-decorated cells were exposed to a CFH concentration series. This was followed by an assay of surviving C3bBb, thus allowing estimation of an IC_50_ value for DAA on a “hostlike” cell surface. Both I62-CFH and V62-CFH exhibited an IC_50_ of ∼1.3 nm in this assay (Fig. 5*A* and Table 2). In a related study, *E*_S_ surfaces were preloaded with C3b and then treated with CFI and a CFH concentration series. The remaining C3b was estimated in a hemolysis assay, as before. This provided an IC_50_ for the CA of CFH on a surrogate self-surface. In this assay, both I62-CFH (IC_50_ = 8.0 nm) and V62-CFH (IC_50_ = 9.5 nm) had similar potencies (Fig. 5*B* and Table 2). The effect of PspCN in both of these assays was explored for I62-CFH. Unexpectedly, I62-CFH alone and I62-CFH–PspCN complex had indistinguishable DAA on *E*_S_-bound C3bBb (Fig. 5*C*). Conversely, and interestingly, PspCN improved the CA of I62-CFH on *E*_S_-bound C3b by a factor of 2 (Fig. 5*D* and Table 2).

**Table 2:** **Decay-accelerating and cofactor activities on sheep erythrocytes**

Experiment	CA IC_50_ (nm)*^a^*	Effect of the variation (or of *PspCN*)*^b^*	DAA IC_50_ (nm)*^a^*	Effect of the variation (or of *PspCN*)*^b^*
I62-CFH *versus*	8.00 ± 0.33	1.19 ± 0.12	1.35 ± 0.10	0.94 ± 0.11
V62-CFH	9.50 ± 0.57		1.27 ± 0.05	
I62-CFH *versus*	8.00 ± 0.33	(0.48 ± 0.03)	1.77 ± 0.05	(0.86 ± 0.05)
I62-CFH + PspCN	3.82 ± 0.06		1.52 ± 0.05	
I62-CFH *versus*	17.5 ± 0.8	2.44 ± 0.26	1.41 ± 0.44	4.72 ± 0.32
R53H-CFH	42.7 ± 2.5		6.65 ± 0.25	
R53H-CFH *versus*	42.7 ± 2.5	(0.40 ± 0.04)	6.68 ± 0.6	(0.91 ± 0.15)
R53H-CFH + PsPCN	17.2 ± 0.6		6.06 ± 0.05	
I62-CFH *versus*	12.0 ± 0.8	>40	1.33 ± 0.09	8.06 ± 1.31
D1119G-CFH	>500		10.7 ± 1.05	
D1119G-CFH *versus*	>500		9.21 ± 0.54	(1.24 ± 0.15)
D1119G-CFH + PsPCN	>500		11.5 ± 0.7	
I62-CFH *versus*	17.5 ± 2.6	3.63 ± 1.35	1.59 ± 0.06	1.83 ± 0.14
LA-CFH	63.4 ± 14		2.92 ± 0.12	
LA-CFH *versus*	63.4 ± 14	(0.75 ± 0.38)	2.23 ± 0.16	(0.87 ± 0.14)
LA-CFH + PsPCN	47.5 ± 13		1.94 ± 0.17	

*^a^* Shown are the S.E. values for the fits of the data from each experiment.

*^b^* The ratio of either IC_50_ for the variant/IC_50_ for I62-CFH or (in parentheses) IC_50_ for CFH with PSPCN/IC_50_ for CFH without PspCN.

In summary, I62-CFH and V62-CFH bound with similar affinities to C3b and to C3d on SPR chips. They had nearly identical DAA on this man-made “foreign” surface. In hemolysis protection assays, both I62-CFH and V62-CFH protected self-like *E*_S_, but neither protected the less self-like *E*_R_ unless PspCN was present. PspCN enhanced binding of both I62-CFH and V62-CFH to C3b and C3d on SPR chips, where it also enhanced DAA. PspCN increased the CA, but not the DAA, of CFH on *E*_S_.

### A disease-linked CCP 1 mutation has similar effects on self-surfaces and foreign surfaces

**Figure 6.**
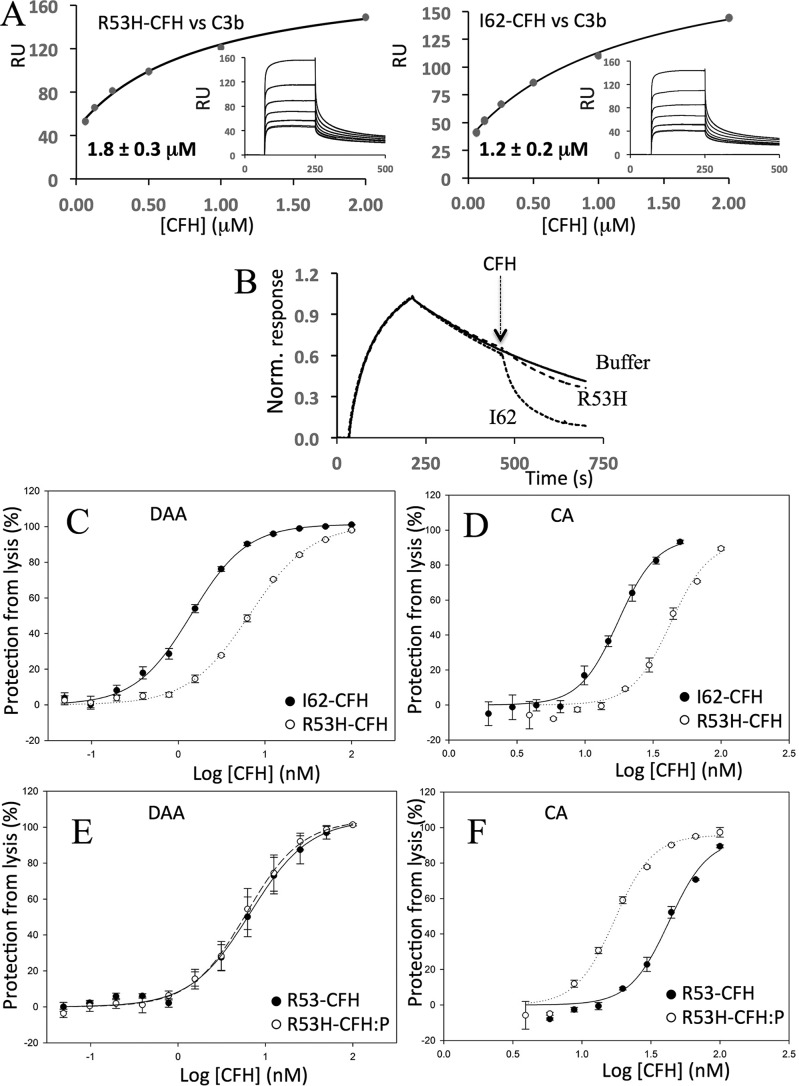
**Effects of R53H on CFH.** *A*, an SPR experiment was performed in a manner similar to that shown in Fig. 2*B* to compare the affinity (for immobilized C3b) of R53H-CFH (*left*) with that of I62-CFH (*right*; see experiment 1 in Table 1). This experiment was repeated with similar results (see experiment 2 in Table 1). *B*, an SPR-based assay of DAA was performed in a manner similar to those shown in Fig. 3 except that 40 nm R53H-CFH or I62-CFH solutions were used. The data shown are representative of two experiments. *C*, an *E*_S_-based assay of DAA was carried out in a manner similar to that shown in Fig. 5*A. D*, an *E*_S_-based assay of CA was carried out in a manner similar to that shown in Fig. 5*B*. Triplicate measurements were made for each data point shown in *C* and *D*, and each experiment was performed once. *E*, in an *E*_S_-surface assay (see Fig. 5*A*), PspCN (mixed 2:1 with R53-CFH) does not restore DAA to R53-CFH. *F*, in an *E*_S_-surface assay (see Fig. 5*B*), PspCN at a 2:1 ratio with R53-CFH enhances its CA. For *C–F*, the mean and S.D. (*error bars*) of triplicate measurements are plotted, and sigmoidal curves were fitted using SigmaPlot. Each graph is representative of two experiments.

The disease-linked R53H mutation in CFH CCP 1 was previously studied within recombinant CFH 1–4 (41). Although R53H did not perturb binding of C3b by CFH 1–4, it did disrupt both the CA and DAA of CFH 1–4 on *E*_S_. Here, we prepared full-length R53H-CFH. In side-by-side comparisons, R53H-CFH bound about as tightly as I62-CFH to amine-coupled C3b: SPR-derived *K_D_* values of 1.2 μm for I62-CFH and 1.8 μm for R53H-CFH (Fig. 6*A* and Table 1) in a first experiment and 1.0 and 1.2 μm for I62-CFH and R53H-CFH, respectively, in a second experiment (Table 1). In these two experiments, PspCN (2:1 molar ratio to R53-CFH) enhanced binding to C3b from 1.8 to 1.0 μm or from 1.0 to 0.6 μm, respectively. This is a PspC-induced gain similar to that seen for I62-CFH (Table 1).

Despite their similar affinities for C3b, R53H-CFH (at 40 nm) exhibited poorer DAA on a CM5-sensor chip (Fig. 6*B*) in comparison with 40 nm I62-CFH. Similarly, in assays of DAA performed on C3bBb-decorated *E*_S_, R53H-CFH (IC_50_ = 6.6 nm) performed less well than I62-CFH (IC_50_ = 1.4 nm) (Fig. 6*C* and Table 2). In an assay on C3b-decorated *E*_S_, R53H-CFH (IC_50_ = 43 nm) was likewise less effective in CA than I62-CFH (IC_50_ = 18 nm) (Fig. 6*D*). PspCN (2:1 molar ratio to CFH) did not noticeably improve the DAA of R53H-CFH measured on *E*_S_ (Fig. 6*E*). A 2-fold ratio of PspCN to R53-CFH did improve its CA on *E*_S_ (Fig. 6*F* and Table 2).

These functional deficiencies of R53H-CFH were not immediately evident in hemolysis protection assays. Although R53H-CFH, with or without PspCN, did not protect foreign *E*_R_ (Fig. 4*D*), it was able to protect the more “self-like” *E*_S_ (Fig. 4*C*). Protection of *E*_S_ in this case may reflect the inability of R53H-CFH to prevent turnover and hence “futile” depletion of C3 in the fluid phase, yet a 1:1 mixture of R53H-CFH with I62-CFH, included in an effort to prevent fluid-phase C3 depletion, was likewise protective in this assay (Fig. 4*E*). By competing with I62-CFH for binding to soluble C3b, R53H-CFH might antagonize the fluid-phase C3-conserving regulatory action of I62-CFH. In a further investigation of this phenomenon, R53H-CFH was mixed 1:1 with the S1191L/V1197A-CFH (LA-CFH) mutant. As described below, LA-CFH prevents C3 depletion in fluid phase, although it exhibits impaired binding to SA-decorated surfaces. The R53H-CFH/LA-CFH mixture was less able to prevent *E*_S_ hemolysis than R53H-CFH alone (Fig. 4*E*). This is consistent with non-depletion of C3, an expected consequence of the fluid-phase regulatory action of LA-CFH. Importantly, it confirms the lack of ability of either LA-CFH or R53-CFH, for different reasons, to fully control C3b amplification on the *E*_S_ surface.

To summarize, R53H-CFH is functionally deficient despite retaining good affinity for C3b. The inability of R53H-CFH to regulate C3b amplification on *E*_S_ is consistent with its poor *E*_S_ hemolysis protection properties under circumstances where fluid-phase C3 levels are not depleted.

### Perturbation of the C-terminal C3b/d-binding site

**Figure 7.**
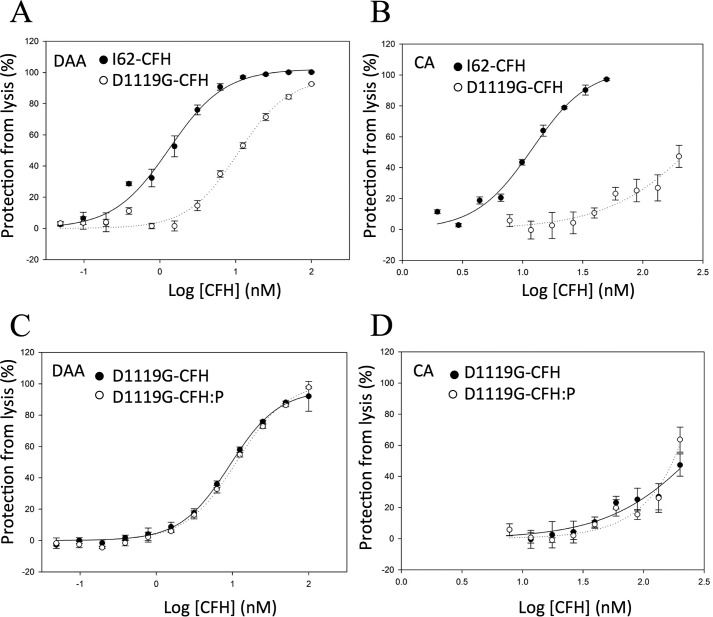
**On erythrocytes, D1119G-CFH has a greater effect on CA than DAA.** In assays similar to the one used to generate Fig. 5 (*A* and *B*), D1119G decreased the DAA of CFH on *E*_S_ (*A*) and devastated CA on *E*_S_ (*B*). *C*, PspCN (mixed 2:1 with D1119G-CFH) had little effect on the DAA of D1119G-CFH measured on *E*_S_. *D*, PspCN (mixed 2:1 with D1119G-CFH) had a small effect on the very low CA of D1119G-CFH measured on *E*_S_. Mean and S.D. (*error bars*) of triplicate measurements are plotted, and sigmoidal curves were fitted using SigmaPlot. Each graph is representative of two experiments.

Asp-1119 of CCP 19 is a key interface residue in the CFH 19-20–C3d complex (53). In recombinant CFH 19-20, D1119G abrogated binding to C3b and C3d (39, 53) and destroyed the ability to compete with plasma CFH for binding to C3b-decorated *E*_S_ (37, 39). In full-length CFH, D1119G was previously shown to degrade performance in *E*_S_ hemolysis protection assays (50). To explore further the functional consequences of D1119G in full-length CFH and allow comparisons with the other CFH mutants reported herein, we measured separately the DAA and CA of D1119G-CFH on *E*_S_. As shown in Fig. 7*A* and Table 2, the ability of D1119G-CFH to accelerate decay of C3bBb on *E*_S_ was diminished compared with wild-type CFH (IC_50_ = 1.3 nm for I62-CFH *versus* IC_50_ = 11 nm for D1119G-CFH). The negative effect of D1119G on the CA of CFH for cleavage of C3b bound to *E*_S_ is greater still (IC_50_ = 12 nm for I62-CFH *versus* IC_50_ > 500 nm for D1119G-CFH) (Fig. 7*B*). PspCN (at a 2:1 molar ratio to D1119G-CFH) had negligible effects on the C3bBb-DAA (Fig. 7*C*) and C3b-CA of D1119G-CFH measured on *E*_S_ (Fig. 7*D*).

### Perturbation of the sialic acid-binding site in CCP 20

**Figure 8.**
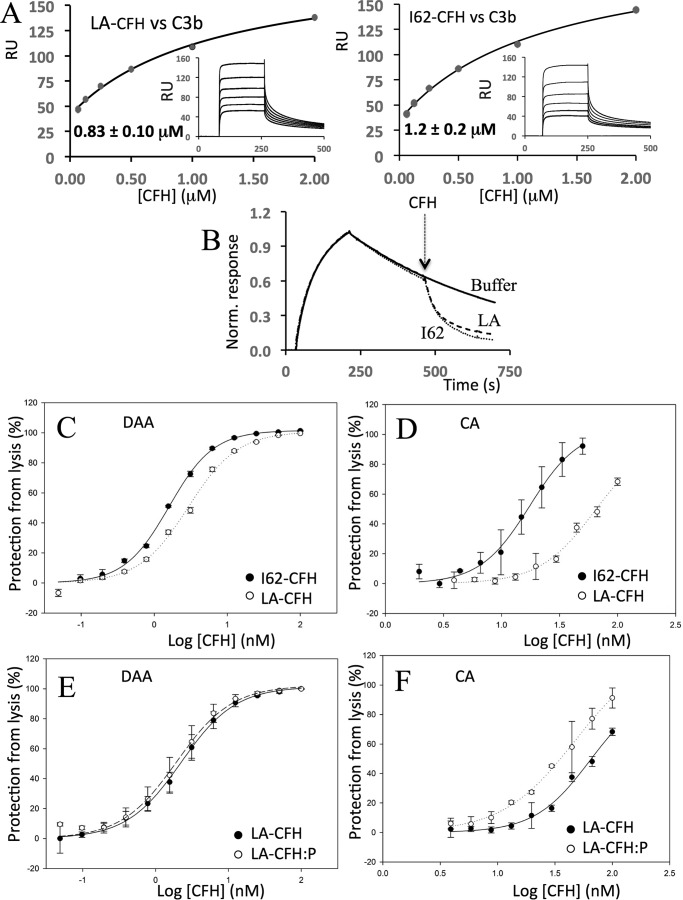
**Effects of S1191L/V1197A on CFH.** *A*, an SPR experiment was performed in a manner similar to that shown in Fig. 2*B* to compare the affinity (for immobilized C3b) of LA-CFH (*left*) with that of I62-CFH (*right*; see experiment 1 in Table 1). This experiment was repeated with similar results (see experiment 2 in Table 1). *B*, an SPR-based assay of the DAA of LA-CFH was performed in a manner similar to those shown in Fig. 3 except that 40 nm CFH solutions were used. The data shown are representative of two experiments. *C*, an *E*_S_-based assay of DAA was carried out in a manner similar to that shown in Fig. 5*A. D*, an *E*_S_-based assay of CA was carried out in a manner similar to that shown in Fig. 5*B. E*, in an *E*_S_-surface assay (see Fig. 5*A*), PspCN (2:1 to LA-CFH) does not restore any DAA to LA-CFH. *F*, conversely, in an *E*_S_-surface assay (see Fig. 5*B*), PspCN (2:1 to LA-CFH) significantly enhanced the CA of LA-CFH. For *C–F*, mean and S.D. (*error bars*) of triplicate measurements are plotted, and sigmoidal curves were fitted using SigmaPlot. Each graph is representative of two experiments.

Ser-1191 and Val-1197 in CCP 20 are critical for recognition of SA according to a crystal structure of a C3d–CFH 19-20–SA complex (54). In previous work, the disease-related double mutation S1191L/V1197A perturbed the ability of CFH 19-20 to compete with full-length CFH for binding to C3b-decorated *E*_S_ (39, 53) but did not perturb the structure of CFH 19-20 nor interfere with its binding, measured by SPR, to C3b or C3d (42). Here, we found that the SPR-measured affinities of full-length LA-CFH and I62-CFH for amine-coupled C3b are indeed similar: *K_D_* = 0.8 μm for LA-CFH *versus* 1.2 μm for I62-CFH in one experiment (Fig. 8*A*) and 0.9 μm for LA-CFH *versus* 1.0 μm for I62-CFH in a second experiment (Table 1). In these two experiments, preincubation of LA-CFH with PspCN in a 2-fold molar excess produced little if any gain in affinity for C3b (*K_D_* = 0.70 μm *versus* 0.83 μm and *K_D_* = 0.60 μm *versus* 0.86 μm, respectively; Table 1). No effect of these mutations on the DAA of full-length CFH, assayed on the SPR chip surface, could be detected (Fig. 8*B*). Taken together, these results suggest that the N-terminal region of CFH dominates its properties on the foreign surface of a SPR chip such that C-terminal mutations have undetectable effects.

When assayed on hostlike *E*_S_ cells, S1191L/V1197A had a smaller effect on DAA than on CA (Fig. 8, compare *C* and *D*). The effect on DAA was measurable but minor (IC_50_ = 1.6 nm for I62-CFH *versus* IC_50_ = 2.9 nm for LA-CFH) (Fig. 8*C* and Table 2), whereas in the CA assays, a larger effect was evident (IC_50_ = 18 nm for IYD-CFH *versus* IC_50_ = 63 nm for S1191L/V1197A-CFH) (Fig. 8*D* and Table 2). Thus, SA binding matters less for DAA than it does for CA. Furthermore, we found that LA-CFH affords significantly less protection than wild-type CFH to *E*_S_ in the hemolysis protection assay (Fig. 4*C*). As is apparent from the results of further experiments on *E*_S_, PspCN did not measurably increase the DAA of LA-CFH, but it clearly enhanced the CA of LA-CFH (Fig. 8, compare *E* and *F*), although the wild-type IC_50_ was not restored (Table 2). In *E*_S_ hemolysis protection assays, the addition of PspCN was, notably, able to partially rescue LA-CFH (Fig. 4*C*), just as it had previously been found to partially rescue another C-terminal mutant, D1119G-CFH (50). Moreover, PspCN also boosted the ability of LA-CFH to protect the foreign surface of *E*_R_ (Fig. 4*D*).

## Discussion

Nearly 100 variations in the human *CFH* gene potentially predispose to aHUS or other diseases (16, 31). Most encode versions of CFH that differ by a single amino acid. Wild-type CFH both inhibits fluid-phase C3 consumption and prevents formation of clusters of deposited C3b molecules on selected surfaces. Only a small fraction of the putatively disease-linked versions of CFH have been functionally characterized in any detail, and 75–80% have not been functionally characterized at all (31). It is often impractical to purify sufficient CFH for rigorous functional analysis from a heterozygous patient. Aiming at a fast and efficient means of obtaining tens of milligrams of protein, we expressed *CFH* variants in *P. pastoris*.

A previously reported careful comparison between plasma-purified versions of V62-CFH and I62-CFH provided a benchmarking opportunity (22). Our yields of recombinant V62-CFH and I62-CFH exceeded those of previous attempts to produce CFH in mammalian (33), insect (34), and moss cells (35, 55). Our versions of V62-CFH and I62-CFH differ in sequence at position 936 as well as at position 62 (*i.e.* we compared V62,E936-CFH with I62,D936-CFH). The E936D SNP is not unique to at-risk or protective haplotypes (20) nor located near binding sites (43) for C3b, C3d(g), or carbohydrates. Although Asp-936 protects against susceptibility to meningococcal disease (56), this SNP is unlikely to impact our assays.

Our recombinant V62-CFH and I62-CFH matched functionally our plasma CFH in our assays of C3b binding, DAA, and CA (Figs. 2–5). Remarkably, however, recombinant V62-CFH and I62-CFH bound very much better than plasma CFH to immobilized C3d (Fig. 2*B*). Our pooled plasma-derived CFH contains a mixture of common haplotypic variants, but these are located in CCPs 1, 7, and 15, so are unlikely to impact directly on C3d binding that involves only CCPs 19 and 20. Contamination with CFH-related proteins in the plasma CFH sample is unlikely because these were removed during purification. Whereas chemical modifications may be present in aging plasma proteins (57), these would be unlikely to have such a drastic and specific effect. The most likely explanation for the difference in C3d binding between recombinant CFH and plasma CFH is the lack of *N*-glycans in recombinant CFH. Natural CFH (36, 58) has eight of these. Some might contribute to the hidden or cryptic nature of the C3d-binding site in CCPs 19 and 20 discussed previously (50).

I62-CFH bound marginally better than V62-CFH to C3b in our SPR-based comparison using identically prepared samples (Table 1), but more replicates would be required to afford significance to this. In further SPR measurements, PspCN enhanced C3b binding and the DAA of I62-CFH and of V62-CFH to nearly identical extents. A slightly tighter complex with C3b formed by I62-CFH was reported in an SPR study conducted on plasma-derived CFH in reverse mode (*i.e.* C3b was flowed over immobilized plasma CFH) (22) (*K_D_* values: Ile-62, 1.04 μm; Val-62, 1.33 μm). Comparisons of I62- and V62-CFH are important because Ile-62 is encoded by an SNP unique to a protective haplotype for AMD and C3G (20). A “complotype” hypothesis (59) predicts that a coincidence of minor functional effects in several complement proteins could be additive in a patient. Our I62-CFH *versus* V62-CFH comparison also agrees with the approximate parity of C3b binding between recombinant Ile-62 and Val-62 variants of CFH 1–4 (41), despite CFH 1–4 binding C3b (*K_D_* = 11–14 μm) much less tightly than full-length CFH.

Reported SPR-derived *K_D_* values for binding of CFH to C3b vary severalfold (36, 49, 53), and the 0.2–0.3 μm *K_D_* values obtained in our three-way comparison are at the tighter end of the range. In other experiments (Table 1), we obtained weaker *K_D_* values, in the 1.0–1.3 μm range, for the same I62-CFH sample. We have, over numerous previous experiments, noticed such variations between SPR-derived *K_D_* values. These probably reflect batch-to-batch variations of C3b or the sensor chip surfaces. Only measurements made on the same chip and in the same experiment can be meaningfully compared.

We mutated another CCP 1 residue (R53H) in CFH and compared the consequences with those reported for R53H in the autonomously functional CFH 1–4 fragment (41, 60). We found (Fig. 6) (i) nearly normal C3b-binding affinity of R53H-CFH; (ii) severalfold diminished complement-regulatory functions of R53H-CFH on *E*_S_; and (iii) negative impacts of R53H on the DAA of CFH on an SPR surface. Encouragingly, these benchmark results closely parallel those obtained for R53H-CFH 1–4.

Such functional deficiencies are consistent with R53H causing aHUS (29), although it should be borne in mind that patients are heterozygous, and serum concentrations of CFH are 1–2 μm (61). Moreover, 250 nm R53H-CFH had wild type–like protective activity in the CFH-reconstituted *E*_s_ hemolysis assay (Fig. 4). To circumvent a potential anomaly of this assay, that fluid-phase C3 depletion arises from lack of control by R53H, we included along with R53H-CFH equimolar amounts of I62-CFH or LA-CFH. I62-CFH regulates complement in fluid phase and on surfaces; LA-CFH regulates complement in fluid-phase only (see below). The 1:1 I62-CFH/R53H-CFH combination, crudely emulating the scenario in heterozygous patients, was also protective in our assay because adequate I62-CFH was present to achieve both fluid-phase and on-surface regulation. Conversely, the 1:1 LA-CFH/R53-CFH was not protective, demonstrating that 250 nm R53-CFH could not protect *E*_S_ from hemolysis if C3 is present in the fluid phase.

Whereas easily prepared N-terminal truncation fragments, such as CFH 1–4, serve as a good model for full-length CFH, the equivalent claim cannot be made for C-terminal fragments. More than 30 substitutions in CCPs 19 and 20 have been linked to aHUS (31). Cross-linking and other evidence suggests that the C-terminal CCPs of CFH modulate the activities carried out by the N-terminal modules of the same molecule (8, 50). Thus, the use of full-length CFH is essential to characterize functional consequences of mutations in CCPs 19 and 20. Aspartate 1119, in CCP 19, is a key residue in the C-terminal C3d and C3b-binding site of CFH (53, 62). In the CFH 19-20 context, an aHUS-linked mutation, D1119G (29), severely reduced binding to C3d and C3b (39, 53). Moreover, D1119G-CFH 19-20 was 40-fold less able than native-sequence CFH 19-20 to compete with CFH for binding to C3b-coated *E*_S_ (39). We previously reported that full-length D1119G-CFH, despite having wild type–like C3b-binding affinity and DAA activity on a foreign SPR chip surface, did not protect *E*_S_ from hemolysis (50). Herein, we have taken this further by showing that D1119G diminishes 10-fold the ability of CFH to accelerate decay of C3bBb assembled on *E*_S_. The effect of D1119G on the CA of CFH, in the *E*_S_ context, is still greater.

We further explored this theme through studies of the aHUS-linked double mutation in CCP 20 of CFH corresponding to a gene conversion with CFHR-1 (*i.e.* S1191L/V1197A) (63). The crystal structure of S1191L-CFH 19-20 bound to C3d is barely distinguishable from that of the wild-type CFH-19-20–C3d complex (42). Conversely, LA-CFH 19-20 failed to bind C3b-decorated *E*_S_ (39). Subsequently, Ser-1191 and Val-1197 were shown to contribute to the primary SA-binding site in the CFH molecule (54). Here, we found that LA-CFH was significantly poorer than wild-type CFH at protecting *E*_S_ from hemolysis by CFH-depleted normal human serum (Fig. 4). A similar result (see above) was obtained using a 1:1 mixture of LA-CFH and R53-CFH, yet in SPR experiments, the S1191L/V1197A substitutions had little impact on binding of CFH to C3b. Moreover, these mutations had only a small effect on C3bBb-DAA whether C3bBb was assembled on an SPR chip surface or, surprisingly, on the surrogate host surface of *E*_S_ (Fig. 8). Conversely, LA-CFH was significantly deficient in CA on *E*_S_ cells. This distinction between DAA and CA, also seen, albeit to a lesser extent, in D1119G, is discussed further below.

PspCN binds plasma CFH tightly, enhancing its binding to C3b and strongly increasing its affinity for C3d. The C3b/C3d(g)-binding and self-recognition site in CCPs 19 and 20 of CFH becomes exposed upon binding PspCN (50), perhaps emulating what happens when CFH binds to a self-surface. Herein, PspCN enhanced the affinities for C3b on an SPR chip, of recombinant I62-CFH, V62-CFH, R53H-CFH, and, to a lesser degree, LA-CFH (Table 1). PspCN also increased, to roughly equal extents, the DAA of I62-CFH and V62-CFH on an SPR chip (Fig. 3). In contrast, PspCN did not increase the DAA of I62-CFH on C3bBb-decorated *E*_S_, and there was a similar lack of responsiveness to PspCN from R53H-CFH, D1119G-CFH, and LA-CFH DAA in the *E*_S_ context (Table 2). The discrepancy between the two assays probably reflects the different surfaces employed: SA-bearing and self-like in the case of *E*_S_ and foreign in the case of SPR. In contrast with its effects on *E*_S_-surface DAA, PspCN significantly enhanced the CA of I62-CFH on C3b-loaded *E*_S_. It likewise had a positive effect, in this context, on the CA of R53H-CFH, and it even partially rescued the CA of LA-CFH (Fig. 8). This difference between the responsiveness to PspCN of DAA and CA on *E*_S_ echoes differences between the impacts, on DAA (small) and CA (larger), of the C-terminal mutations S1191L/V1197A and, to a less pronounced extent, D1119G (Table 2).

Both DAA and CA reside in CFH CCPs 1–4 and can act in a concerted manner, but each involves a distinct sequence of events (Fig. 1). To accelerate C3bBb decay, CFH probably binds both the surface-attached C3b and the Bb (64) components of C3bBb, whereupon it acts catalytically to dissociate C3bBb into C3b and Bb. Following release of Bb, surface-tethered C3b retains affinity for CFH because both binding sites on C3b, engaged by CFH CCPs 1–4 and CCPs 19 and 20, remain intact, as do interactions between CCP 20 and SA on the surface. The CFH molecule must subsequently dissociate from C3b to catalyze another C3bBb decay event. As a cofactor, CFH binds to surface-attached C3b and recruits CFI (65). Subsequent cleavage of C3b to iC3b destroys its binding site for CFH CCPs 1–4 (66). Importantly, the binding site on iC3b used by CCPs 19 and 20, along with the interaction between CCP 20 and SA, is not affected. The presence of the product, iC3b or C3d(g), on an SA-decorated surface thus creates the possibility of retaining or enriching CFH on that surface by binding to the CFH C terminus. Thus retained, these CFH molecules could utilize their CCPs 1–4 to act as a cofactor on nearby C3b molecules. In short, complete detachment and reattachment of CFH from the surface is likely between cycles of DAA, but in the case of CA, CFH can cycle between iC3b(C3d)-bound, iC3b(Cd)-plus-C3b-bound, and C3b-bound forms, without leaving the surface.

The results obtained here for disease-linked C-terminal mutants on *E*_S_ surfaces support this line of reasoning because they show that retention of CFH on a surface via its CCPs 19 and 20 is more important for CA than for DAA. Our results with PspCN likewise support this reasoning because they confirm that the PspCN-induced increase in access to the surface-engaging C-terminal modules has little effect on DAA but a positive effect on CA.

To summarize, our results show that acceleration of C3bBb decay on a hostlike surface is not very responsive to the presence or the absence (as tested by mutagenesis (Fig. 8*C*)) or the availability or unavailability (tested by the inclusion or not of PspCN (Fig. 8*E*)) of the self-recognition site in CCPs 19 and 20. On the other hand, CA (for CFI on C3b bound to *E*_S_) is sensitive to the presence or absence (mutagenesis) of the self-recognition site (Fig. 8*D*) and its availability as enhanced by PspCN (Fig. 8*F*).

Soluble PspCN has a protective effect on *E*_R_ in hemolysis assays (Fig. 4). This was observed previously and is consistent with bacterially displayed PspC preventing C3b amplification on the microbial surface. PspCN also partially rescued functionally deficient LA-CFH in an *E*_S_ hemolysis protection assay. These are more complex assays (compared with the individual CAA and DAA assays) and less straightforward to interpret. Nonetheless, we infer that PspCN can work as a protector of self-surfaces mainly by enhancing CFH CA on these surfaces, and this suggests new therapeutic strategies based on CFH activators.

## Experimental procedures

### Preparation of proteins

Human CFH was either purchased from Complement Technologies or purified from pooled plasma (TCS Biosciences Ltd.). Other plasma-derived human complement proteins (CFB, CFD, CFI, C3b, and C3d) and CFH-depleted normal human serum (Complement Technologies) were stored as recommended by the supplier and used without further purification.

Recombinant human CFH, corresponding to allotypic variant, Val-62, Tyr-402, Glu-936 (V62-CFH), was produced and purified, following treatment with EndoH_f_ to trim *N*-glycans to GlcNAc residues, as described (36). Preparation of the D1119G-CFH mutant (on a Val-62 background) was described previously (50). Similar strategies were used to produce variant Ile-62, Tyr-402, Asp-936 (I62-CFH) along with R53H-CFH and S1191L/V1197A-CFH on the Ile-62 background. In brief, expression-optimized DNA encoding the target protein sequence was synthesized (GeneArt-Life Technologies) and subcloned into *Pichia pastoris* expression vector pPICZaB (Invitrogen). This was amplified in XL1 Blu *Escherichia coli* cells (Agilent) and then used to transform the KM71H strain of *P. pastoris* (Invitrogen). Single colonies grown on yeast-peptone-dextran (YPD) agar plates with 300 μg/ml Zeocin were picked. The colony with the highest level of CFH production in small-scale trials was used to inoculate a BioFlow 4500 (New Brunswick Scientific) 10-liter fermenter. After a glycerol-fed batch phase, protein production was induced by a 4-day methanol/glycerol continuous feed. Secreted proteins were chromatographically purified (36) after enzymatic deglycosylation.

The protein PspCN was produced as described (50). In brief, a synthetic gene encoding positions 37–140 of PspC from D39 *Streptococcus pneumoniae* was expression-optimized for *E. coli* (GeneArt-Life Technologies). It was cloned into *E. coli* vector pE-SumoPro-Kan (LifeSensors, Malvern, PA) and then expressed in BL21 (DE3) *E. coli* cells (Lucigen). The resultant N-terminally His_6_-SUMO-tagged protein was captured on nickel-affinity resin, eluted with imidazole, and then further purified by cation-exchange chromatography. The His_6_-SUMO tag was cleaved from the fusion protein using ULP1, a SUMO-specific protease, and removed by re-passing over the nickel-affinity resin. Cleaved material was purified by size-exclusion chromatography. Homogeneity was confirmed by SDS-PAGE and MS.

### Measurement of CFH and CFH–PspCN binding to C3b by SPR

Experiments were performed on a Biacore T200 at 25 °C as described previously (43). Briefly, C3b was attached using standard amine coupling to flow cells of a Biacore C1 chip (GE Healthcare). A second flow cell was used for background subtraction. A dilution series of CFH solutions in 10 mm HEPES buffer, 150 mm NaCl, 0.05% (v/v) surfactant P20 (GE Healthcare), preincubated with or without a 2-fold molar excess of PspCN, was injected for 150 s at 50 μl/min and a dissociation time of 600 s. The sensor chip was regenerated by three injections of 1 m NaCl. Data were analyzed using the Biacore Evaluation software and a 1:1 steady-state binding model.

### C3bBb decay-accelerating assay

Decay-accelerating activity was measured in an SPR-based assay as described (67). In brief, ∼500 RU of C3b were attached to a Biacore CM5-sensor chip (GE Healthcare) using standard amine coupling. Subsequently, C3bBb was assembled on the chip by a 180-s 10-μl/min injection of a solution containing 500 nm CFB and 50 nm CFD. Formation of C3bBb (∼150 RU) was monitored, and then an initial dissociation phase (of 240-s duration) allowed observation of intrinsic convertase decay (*i.e.* a decline in RUs attributable to loss of Bb). Subsequently, CFH or CFH–PspCN was injected (10 μl/min for 180 s), allowing observation of a further dissociation phase. The chip was regenerated between cycles by a 30-s injection of 0.1 μm CFH and three 30-s injections of 1 m NaCl.

### Preparation of cells for complement regulation assays

According to Tortajada *et al.* (22), *E*_S_ in Alsever's solution (TCS Biosciences) were pelleted and resuspended three times in complement-fixation diluent (C-FxD) (Oxoid, Thermo Fisher Scientific). The washed cells, at 2% (w/v) in C-FxD, were sensitized by incubation with anti-*E*_S_ stroma antibody from rabbit (Sigma) (diluted 1:4000 (v/v) in the C-FxD) for 30 min at 37 °C. Sensitized sheep erythrocytes (*EA*_S_) were washed three times in C-FxD and then resuspended in C-FxD to a final cell density of 2% (w/v). C3b was then deposited on the *EA*_S_ surface by adding one volume of 4% (v/v) CFB-deficient and CFH-deficient normal human serum (ΔBΔH-NHS) (22) containing 6 μg/ml *Ornithodoros moubata* complement inhibitor (a gift from Varleigh Immuno Pharmaceuticals) in C-FxD, followed by a 20-min incubation at 37 °C. The C3b-coated *EA*_S_ (*EA*_S_-C3b) were washed three times in AP buffer (5 mm sodium barbitone, pH 7.4, 150 mm NaCl, 7 mm MgCl_2_, 10 mm EGTA). Finally, *EA*_S_-C3b cells were resuspended at 2% (v/v) in AP buffer.

### Cofactor assays on erythrocytes

Cofactor activity was determined by adding (in triplicate) 50 μl from a dilution series of wild-type or mutant CFH solutions, with or without PspCN at a 2:1 PspCN/CFH molar ratio, to 50 μl of a 2% (w/v) suspension of *EA*_S_-C3b in AP buffer containing 2.5 μg/ml CFI. After 15 min (25 °C) of C3b proteolysis, cells were washed three times in 200 μl of AP buffer and pelleted. Washed cells were resuspended in 50 μl of AP buffer in preparation for C3bBb formation as an indirect measure of surviving C3b (22). To this end, C3bBb was formed by adding 50 μl of a solution containing 40 μg/ml CFB and 0.2 μg/ml CFD to 50 μl of the CFH-treated and CFI-treated *EA*_S_-C3b and incubating for 15 min at 37 °C. Lysis was then used as a read-out of C3bBb formed on cells. This part of the assay was performed by adding ΔBΔH-NHS to a final concentration of 4% (v/v) to the suspended cells and incubating for 20 min at 37 °C. To quantify lysis, cells were pelleted, and the absorbance (*A*_420 nm_) of the supernatant was measured on a Multiskan Ascent Plate Reader (Agilent). The no-lysis controls, for blank subtraction, contained no CFB or CFD. Maximal lysis was determined by an *A*_420_ measurement following the addition of PBS, instead of CFH solution, to the EA_S_-C3b. Mean readings from experiments performed in triplicate were calculated. Total protection from lysis for each concentration of CFH was determined by subtracting, from 1, the following: ((mean *A*_420_ for a particular CFH sample) − (mean *A*_420_ for a no-lysis control sample))/(A_420_ for a sample where PBS was used rather than CFH). This was then multiplied by 100. A sigmoidal curve was fitted using SigmaPlot and employed to determine the concentration at which CFH gave 50% protection from lysis (IC_50_).

### Decay-accelerating activities on erythrocytes

For these assays, C3bBb was formed on *EA*_S_-C3b by adding 50 μl of AP buffer containing 40 μg/ml CFB and 0.2 μg/ml CFD to 50 μl of a cell suspension (22). After 15 min (37 °C), C3bBb formation was stopped by the addition of PBS containing 250 μm EDTA, pH 7.4, at a 1:20 dilution. Following the addition of 50 μl from a dilution series of (wild-type or mutant) CFH solutions (with or without PspCN at a 2:1 ratio) in PBS, C3bBb was allowed to decay for 15 min (25 °C). The extent of lysis then served as a measure of remaining C3bBb, as described above. As above, mean readings of experiments in triplicate were calculated. Total protection from lysis for each concentration of CFH was determined as above, and a sigmoidal curve was fitted using SigmaPlot to determine the concentration at which CFH gave 50% protection from lysis (IC_50_).

### Hemolysis protection assays

For this 96-well plate assay (46), *E*_S_ and *E*_R_ (TCS Biosciences) were prepared by washing three times with buffer E1 (20 mm HEPES, 145 mm NaCl, 0.1% (w/v) gelatin (porcine skin; Fluka), 10 mm EDTA, pH 7.3) and then buffer E2 (20 mm HEPES, 145 mm NaCl, 0.1% (w/v) gelatin, 10 μm EDTA, pH 7.3). A stock of *E* was suspended in E2 at a density such that an *A*_412_ nm = 1.0–1.2 resulted following complete lysis in water. CFH-depleted serum (ΔH-NHS) was reconstituted with a stated, final concentration (250 nm to 1.0 μm) CFH, with or without PspCN (at a 2:1 PspCN/CFH ratio), and then aliquots of varying size were combined, to give various percentages of ΔH-NHS/CFH, with 5 μl of *E* stock and buffer E2 to a final volume of 45 μl. Lysis was initiated by adding 5 μl of 50 mm MgEGTA, pH 7.3. The reaction was incubated (37 °C) for 30 min and then quenched with 200 μl of buffer E1. The plate was spun (1500 × *g*, 10 min, 4 °C) to pellet non-lysed cells, and then 100 μl of supernatant from each well was transferred to another 96-well plate for *A*_412_ measurements.

## Author contributions

H. K. produced the CFH mutants and carried out some of the assays; E. W. performed the erythrocyte-based assays of CA and DAA; E. M. contributed to the production, and carried out the SPR-based comparisons, of V62-CFH and I62-CFH; Y. Y. carried out the hemolysis protection assays; K. M. designed experiments and contributed to writing the manuscript; D. K. and A. R. helped to conceive the study and contributed to the experimental design; A. P. H. contributed to the experimental design and to the production and purification of proteins; P. N. B. helped to conceive the study, contributed to the experimental design, and wrote the manuscript.

## Footnotes

3 The abbreviations used are:

CFB: CFD, CFH, and CFI, complement factor B, D, H, and I, respectively
aHUS: atypical hemolytic uremic syndrome
AMD: age-related macular degeneration
C3G: C3 glomerulopathy
CA: cofactor activity
CCP: complement-control protein module
C-FxD: complement-fixation diluent
DAA: decay-accelerating activity
*E*_R_: rabbit erythrocytes
*E*_S_: sheep erythrocytes
*EA*_S_: sensitized sheep erythrocytes
GAG: glycosaminoglycan
LA-CFH: S1191L/V1197A-CFH
NHS: normal human serum
SA: sialic acid
PspCN: *S. pneumoniae* choline-binding protein, N-terminal domain
SPR: surface plasmon resonance
CFHR: CFH-related protein
RU: resonance units.
